# Reevaluating the Genetic Contribution of Monogenic Dilated Cardiomyopathy

**DOI:** 10.1161/CIRCULATIONAHA.119.037661

**Published:** 2020-01-27

**Authors:** Francesco Mazzarotto, Upasana Tayal, Rachel J. Buchan, William Midwinter, Alicja Wilk, Nicola Whiffin, Risha Govind, Erica Mazaika, Antonio de Marvao, Timothy J.W. Dawes, Leanne E. Felkin, Mian Ahmad, Pantazis I. Theotokis, Elizabeth Edwards, Alexander Y. Ing, Kate L. Thomson, Laura L.H. Chan, David Sim, A. John Baksi, Antonis Pantazis, Angharad M. Roberts, Hugh Watkins, Birgit Funke, Declan P. O’Regan, Iacopo Olivotto, Paul J.R. Barton, Sanjay K. Prasad, Stuart A. Cook, James S. Ware, Roddy Walsh

**Affiliations:** 1 National Heart and Lung Institute (F.M., U.T., R.J.B., W.M., A.W., N.W., R.G., E.M., T.J.W.D., L.E.F., M.A., P.I.T., E.E., A.J.B., A.M.R., P.J.R.B., S.K.P., S.A.C., J.S.W.), Imperial College London, United Kingdom.; 2 Medical Research Council-London Institute of Medical Sciences (N.W. A.d.M., T.J.W.D., D.P.O., S.A.C., J.S.W.), Imperial College London, United Kingdom.; 3 Cardiovascular Research Centre, Royal Brompton and Harefield National Health Service Foundation Trust, London, United Kingdom (F.M., U.T., R.J.B., W.M., A.W., N.W., R.G., E.M., L.E.F., M.A., P.I.T., E.E., A.J.B., A.A.P., A.M.R., P.J.R.B., S.K.P., S.A.C., J.S.W.).; 4 Cardiomyopathy Unit, Careggi University Hospital, Florence, Italy (F.M., I.O.).; 5 Department of Experimental and Clinical Medicine, University of Florence, Italy (F.M., I.O.).; 6 Laboratory for Molecular Medicine, Partners HealthCare Personalized Medicine, Cambridge, MA (A.Y.I.).; 7 Oxford Medical Genetics Laboratory, Oxford University Hospitals National Health Service Foundation Trust, The Churchill Hospital, United Kingdom (K.L.T.).; 8 Radcliffe Department of Medicine, University of Oxford, United Kingdom (K.L.T., H.W.).; 9 National Heart Centre Singapore (L.L.H.C., D.S., S.A.C.).; 10 Department of Pathology, Massachusetts General Hospital and Harvard Medical School, Boston (B.F.).; 11 Duke-National University of Singapore Medical School (S.A.C.).; 12 Department of Clinical and Experimental Cardiology, Heart Center, Amsterdam Cardiovascular Sciences, Amsterdam Universitair Medische Centra, University of Amsterdam, The Netherlands (R.W.).

**Keywords:** dilated cardiomyopathy, ExAC, genetic testing, Mendelian genetics, rare variant association testing

## Abstract

Dilated cardiomyopathy (DCM) is genetically heterogeneous, with >100 purported disease genes tested in clinical laboratories. However, many genes were originally identified based on candidate-gene studies that did not adequately account for background population variation. Here we define the frequency of rare variation in 2538 patients with DCM across protein-coding regions of 56 commonly tested genes and compare this to both 912 confirmed healthy controls and a reference population of 60 706 individuals to identify clinically interpretable genes robustly associated with dominant monogenic DCM.

**Methods:**

We used the TruSight Cardio sequencing panel to evaluate the burden of rare variants in 56 putative DCM genes in 1040 patients with DCM and 912 healthy volunteers processed with identical sequencing and bioinformatics pipelines. We further aggregated data from 1498 patients with DCM sequenced in diagnostic laboratories and the Exome Aggregation Consortium database for replication and meta-analysis.

**Results:**

Truncating variants in *TTN* and *DSP* were associated with DCM in all comparisons. Variants in *MYH7, LMNA, BAG3, TNNT2, TNNC1, PLN, ACTC1, NEXN, TPM1*, and *VCL* were significantly enriched in specific patient subsets, with the last 2 genes potentially contributing primarily to early-onset forms of DCM. Overall, rare variants in these 12 genes potentially explained 17% of cases in the outpatient clinic cohort representing a broad range of adult patients with DCM and 26% of cases in the diagnostic referral cohort enriched in familial and early-onset DCM. Although the absence of a significant excess in other genes cannot preclude a limited role in disease, such genes have limited diagnostic value because novel variants will be uninterpretable and their diagnostic yield is minimal.

**Conclusions:**

In the largest sequenced DCM cohort yet described, we observe robust disease association with 12 genes, highlighting their importance in DCM and translating into high interpretability in diagnostic testing. The other genes analyzed here will need to be rigorously evaluated in ongoing curation efforts to determine their validity as Mendelian DCM genes but have limited value in diagnostic testing in DCM at present. This data will contribute to community gene curation efforts and will reduce erroneous and inconclusive findings in diagnostic testing.

## Abstract 2

Supplemental Digital Content is available in the text.

**Clinical Perspective**

**What Is New?**

- This is the largest genetically characterized cohort of patients with dilated cardiomyopathy (DCM) yet described, comprising 2538 probands.
- By comparing the burden of rare variation in DCM cases with a cohort of healthy controls and reference population data, we demonstrate that most genes implicated in DCM do not have a significant enrichment of rare variants in cases, indicating they are likely to be, at best, rarely causative.
- By analyzing 2 DCM cohorts with distinctive patient profiles, we are able to comprehensively evaluate the genetic basis of DCM and identify variant classes that are particularly associated with early onset disease.

**What Are the Clinical Implications?**

- This study identifies the genes and variant classes that are most relevant for DCM genetic testing and those likely to yield interpretable results.
- Our findings will provide key evidence for curation efforts such as the ClinGen initiative that will define valid disease genes for DCM.
- By restricting analysis to validated and interpretable genes and variant classes, we can increase the accuracy and reduce the uncertainty associated with clinical genetic testing in DCM.

Dilated cardiomyopathy (DCM) is an inheritable heart disease affecting up to 1 in 250 individuals and is characterized by genetic heterogeneity, and variable penetrance and expressivity.^1^ The advent of efficient high-throughput sequencing platforms has led to a rapid increase in the number of genes in which variants have been reported as causative, largely based on candidate-gene approaches. The Human Gene Mutation Database collated 68 genes as associated with primary DCM between 1996 and 2015, and >100 genes are now routinely tested in clinical diagnostic laboratories, including those implicated in syndromic forms.^2^ Variant interpretation in these genes is a key challenge faced by clinicians and geneticists.

However, previous studies often did not adequately control for background population variation, especially before large reference population datasets were available. The release of the Exome Aggregation Consortium (ExAC) database showed rare variants to be collectively common in the population—with each individual carrying ~54 variants previously reported as pathogenic^3^—and provides the opportunity to reappraise putative genetic associations by comparing variant prevalence in patient cohorts with the >60 000 individuals in ExAC. We recently demonstrated the potential of this approach through analysis of genes implicated in DCM^4^ and hypertrophic cardiomyopathy.^4,5^ However, the DCM study was limited by the use of patients referred for genetic testing, which introduces important bias (as testing is particularly recommended for young and familial patients, and for clinical subphenotypes with specific genetic associations, such as *LMNA* cardiomyopathy), by the limited number of cases with data for some genes (range, 121–1315 individuals per gene), and by the absence of data for recently implicated genes such as *ZBTB17*^6^ and *BAG3.*^7^ Here we address these limitations by providing comprehensive analysis of 2538 patients with DCM from 2 distinct cohorts—a prospectively ascertained adult outpatient cohort and an expanded diagnostic laboratory referral cohort—sequenced in up to 56 genes. Notably, our previous work encompassed the analysis of 22 140 sequenced patient-gene pairs, whereas here we analyze 89 463. This work represents a critical appraisal of the importance of genes implicated in monogenic dominant DCM, and a reappraisal of its genetic architecture to aid accurate identification of pathogenic variants, minimize ambiguous and erroneous findings, and inform genetic testing strategies.

## Methods

All data and analytic methods for this study are available to other researchers for purposes of reproducing the results or replicating the procedure (Methods and Tables in the online-only Data Supplement).

### Study Population

#### Primary Cohorts

The DCM primary outpatient clinic cohort consisted of 1040 patients with DCM: 863 recruited to the National Institute for Health Research Biobank at the Royal Brompton Hospital, London (15 of 863 younger than 18 years) and 177 to the Singapore Biobank at the National Heart Centre Singapore. All patients were prospectively enrolled for research purposes and underwent cardiac phenotyping with either cardiovascular magnetic resonance (CMR) or transthoracic echocardiography, with DCM diagnosed according to standard criteria (described in Notes in the online-only Data Supplement).

The primary control cohort was composed of 912 healthy controls recruited prospectively via advertisement for the UK Digital Heart Project at the Medical Research Council London Institute of Medical Sciences and Imperial College London.^8^ These individuals had no history of medical illness and negative family history for cardiovascular disease, were not taking regular medication, and did not have evidence of cardiac structural or functional impairment on CMR imaging. Primary cases and controls underwent identical sequencing and bioinformatics data processing on the TruSight Cardio panel^9^ (details in Notes in the online-only Data Supplement). All participants gave written informed consent, and the study was approved by the relevant regional research ethics committees. Demographic and clinical characteristics of primary DCM cases and controls are provided in Table I in the online-only Data Supplement.

#### Secondary DCM Cohort From Clinical Genetic Testing

**Figure 1.**
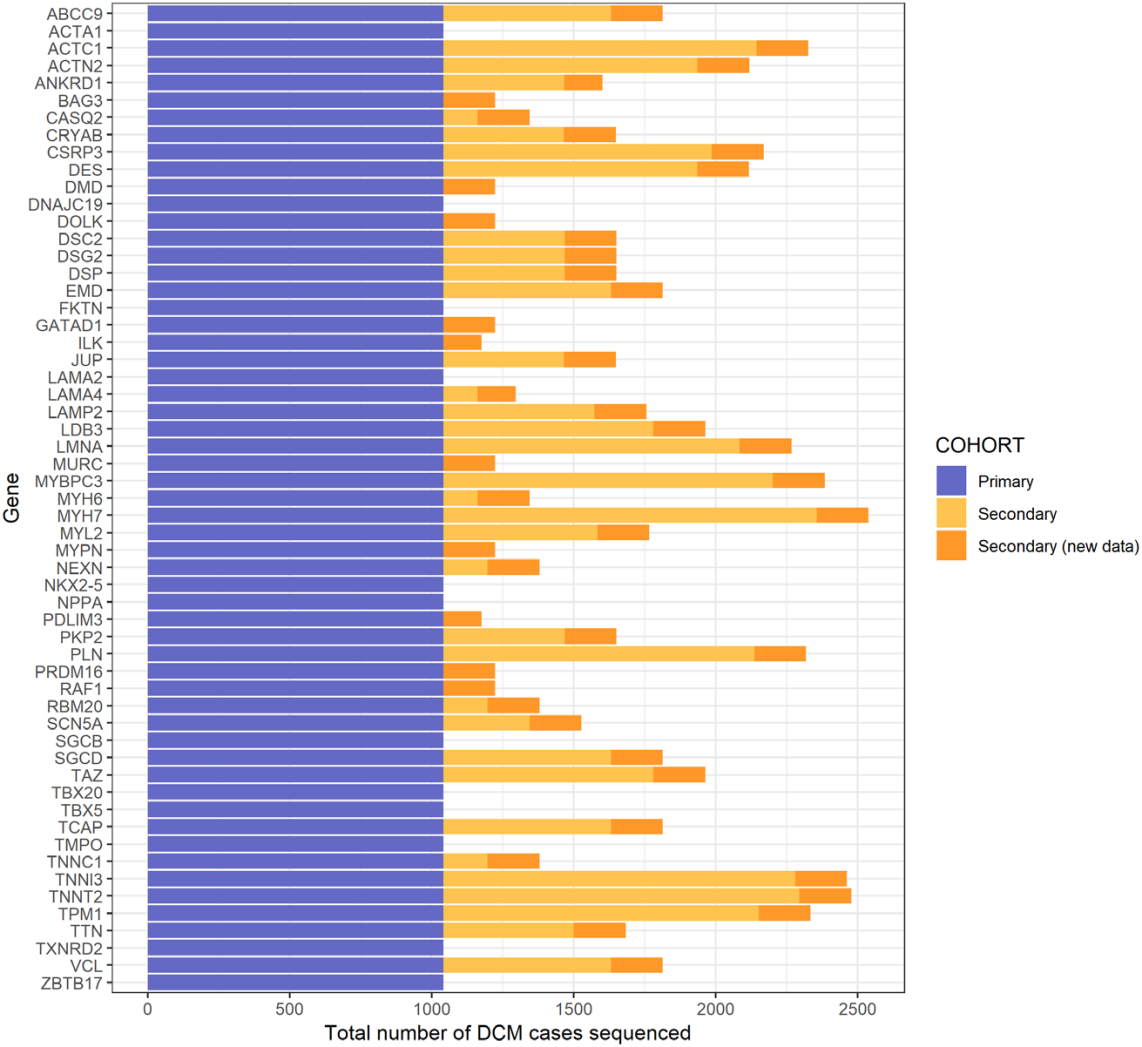
**Number of dilated cardiomyopathy (DCM) cases sequenced for each gene and cohort.** The primary cohort is the outpatient DCM cohort. The secondary clinical genetic testing referral cohort was composed of cases that had been previously reported^4^ (light orange) along with data reported here for the first time (dark orange).

The secondary diagnostic referral cohort was composed of 1498 patients referred for clinical genetic testing to either the Oxford Medical Genetics Laboratory (up to 22 genes sequenced in 304–498 patients) or the Laboratory of Molecular Medicine (up to 45 genes sequenced in 135–939 patients). A proportion of this cohort has been previously reported,^4^ here supplemented with new data from 183 additional cases and 10 additional genes (Figure 1 and Table II in the online-only Data Supplement). Age at diagnosis was available for 691 patients, of whom 286 were younger than 18 years (Figure I in the online-only Data Supplement).

#### Secondary Reference Population

The ExAC dataset is a collation of whole-exome sequencing cohorts reprocessed through the same bioinformatics pipeline, numbering 60 706 unrelated individuals. We used ExAC as a reference population to define rare variants, and as a secondary control dataset, as maximizing cohort dimensions is key to assess the overall variation burden in small and highly constrained genes. Although not healthy controls per se, the ExAC subcohorts are from studies of non-Mendelian diseases and population controls that are not expected to be enriched for DCM cases, and with precautions, ExAC is therefore a suitable control for gene-centric analyses of Mendelian disease.^10^

### Selection of Relevant DCM Genes

Fifty-six genes with ≥1 variant reported as pathogenic for DCM between 1996 and 2015 in the Human Gene Mutation Database (professional v2015.3) and included on the Illumina TruSight Cardio panel^9^ were selected for inclusion in the analysis, with 44 also targeted by the clinical panels used at the Laboratory of Molecular Medicine and Oxford Medical Genetics Laboratory (Table II in the online-only Data Supplement). Of note, 22 of these 56 genes were not sequenced in our previously published diagnostic laboratory cohorts.^4^ Although sequenced in all cohorts, *RBM20* was excluded from analysis because of suboptimal sequencing coverage in ExAC (Figure II in the online-only Data Supplement), which is likely to confound rare variant identification.

### Burden Testing

The combined frequency of rare variants altering canonical transcripts of each gene was calculated in each cohort (transcript details in Table II in the online-only Data Supplement). Rare is defined by a minor allele frequency <0.0001 in ExAC, to allow these data to be directly comparable with previous analyses.^4,5^ Only protein-altering variants were compared, with separate calculations for predicted protein-truncating (nonsense, frameshift, and essential splice site) and nontruncating variation (missense and inframe indels). In *TTN*, only variants altering exons constitutively expressed in the heart (spliced into >90% of the cardiac transcripts, Percent Spliced In>90%)^11^ were included in our analysis. Details of all variants are shown in Tables III, IV, V, VI, and VII in the online-only Data Supplement.

A number of steps were taken to ensure we could confidently compare exome sequencing data from ExAC with the targeted panel data from the primary cohorts.

First, we controlled for the variable sequencing coverage of each gene in ExAC by adjusting to effective population size to reflect the proportion of ExAC participants in whom the gene was adequately covered (ie, with a PASS filter value, an average per-base genotype quality score ≥20 and read depth ≥10×).

Second, we developed an algorithm to calibrate variant-site quality filters based on the framework recently proposed by Guo et al,^10^ iteratively comparing the prevalence of rare synonymous variants—not expected to be disease-related—between targeted panel data (joint primary DCM and healthy control cohorts) and whole-exome sequencing data from ExAC with different cut-off combinations. Once the calibration parameters ensuring equal variant detection in panel data and ExAC were detected (ie, no significant burden differences between panel data and ExAC at the single gene level as well as considering all 56 genes together, and genomic inflation factor between 0.95 and 1.05), the same cut-offs were applied to all subsequent comparisons on protein-altering variants. Details are provided in the Notes in the online-only Data Supplement.

Third, because our analysis also includes ExAC as a control cohort to maximize statistical power, we compared variant burdens in ExAC to those in the healthy control cohort.

**Figure 2.**
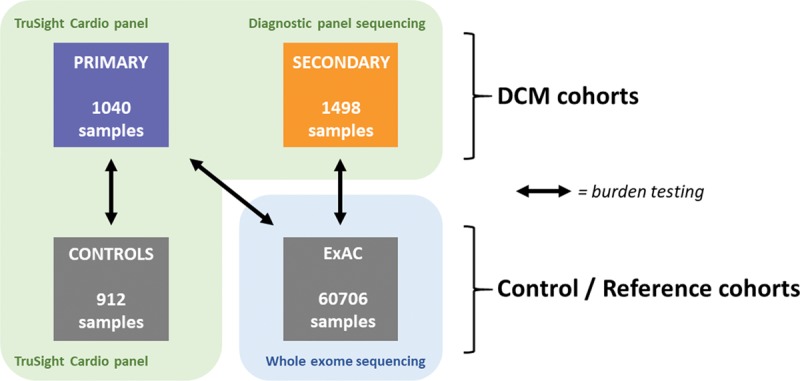
**Overview of the cohorts and rare variant burden analyses performed in this study.** The primary cohort (n=1040) was compared with 912 healthy controls sequenced on the same platform and with the Exome Aggregation Consortium (ExAC) reference dataset. A further 1498 cases were sequenced in diagnostic laboratories on a range of platforms over a decade, and compared with ExAC (n=60 706). Comparing both cohorts with the ExAC reference population dataset, extensive quality control was performed to minimize differences caused by sequencing technologies.

The burden of rare protein-altering variants at the single-gene level in cases and controls was assessed over 3 comparisons: primary DCM cases vs healthy controls sequenced on the same platform, primary DCM cases vs ExAC, and secondary DCM cases vs ExAC (Figure 2).

Because different ethnicities were included, an additional sensitivity comparison was performed downstream between self-reported white patients with DCM and the Non-Finnish European subset of ExAC to demonstrate that results were not driven by stratification bias and consistent results are observed when assessing all ethnicities and whites only.

### Statistical Analysis

Burden comparisons were performed using Fisher’s exact test (1-sided for testing enrichment, ie, DCM cases vs healthy controls and vs ExAC; and 2-sided for testing differences, ie, healthy controls vs ExAC and comparisons of synonymous variants burden). All reported *P* values are corrected with the Bonferroni method for testing 56 genes (nominal P*56) unless otherwise specified, so a *P*<0.05 is considered significant. Correlation of burden differences in the sensitivity analysis was measured using Pearson’s R coefficient.

Age at diagnosis was compared between genotype-positive (ie, carriers of ≥1 variant of any variant class enriched in DCM) and genotype-negative individuals by means of a Wilcoxon rank-sum test, and among the different variant classes using a Kruskal-Wallis test.

### Etiological Fraction

For each variant class with a significant excess in DCM cohorts, we computed the corresponding etiological fraction, a derivative of the odds ratio (OR)


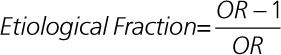


which provides a quantitative measure of the interpretability of such variants.^4^ The etiological fraction estimates the proportion of confirmed cases with rare variants in which the disease is attributable to the presence of the rare variant itself. In a dominant monogenic inheritance model, this also represents prior probability that a rare variant is pathogenic, given that it is found in a confirmed case.

### Statistical Power

Comparing 1040 patients with DCM with 912 healthy controls gives 80% power to detect a burden difference of 1% against a background variation rate of 0.36% in controls for a single gene tested. Using ExAC as a control cohort (mean size = 58 666 individuals sequenced for the genes analyzed) increases sensitivity, allowing detection of a 1% difference with a background control variation rate ~4-fold higher (1.22%). Details on how these calculations were performed are provided in the Notes in the online-only Data Supplement.

## Results

### Technical Quality Control and Suitability of ExAC as Control Cohort

As expected, coverage in ExAC (ie, mean percentage of sample bases covered at 10×) was significantly lower and more variable compared with our targeted panel data (ExAC: 88.3±11.3%, panel: 99.8±0.3%, *P*=9.4×10^–19^; Figure II in the online-only Data Supplement). Controlling for the variable sequencing coverage and applying the quality control steps outlined in the Methods, the frequency of rare (minor allele frequency <0.0001) synonymous variants in the 56 genes tested was comparable between primary cohorts (n=1040 DCM + 912 healthy controls) and ExAC across the gene set (31.6% in panel data vs 30.3% in ExAC, *P*=0.25) and in single genes (0.65≤*P*≤1, Figure III and Table VIII in the online-only Data Supplement), confirming comparability of sequencing data from primary cohorts and ExAC in terms of variant detection sensitivity. The corresponding optimal genomic inflation factor for the distribution of the 56 single-gene *P* values was 1.008 (Figure IV in the online-only Data Supplement), ensuring the absence of systematic bias.

Applying the derived optimal variant quality cut-offs, burden testing of rare protein-altering variants showed comparable variant frequencies in the 912 confirmed healthy controls and ExAC at the gene-set level (67.7% in healthy controls vs 67.8% in ExAC, *P*=0.94) as well as at the single-gene level (0.07<*P*<1, Figure V and Table IX in the online-only Data Supplement), confirming the suitability of ExAC as control cohort when appropriate filtering is applied.

### Burden of Rare Protein-Altering Variants in DCM Compared With Control Cohorts

#### DCM Primary Outpatient Cohort

**Figure 3.**
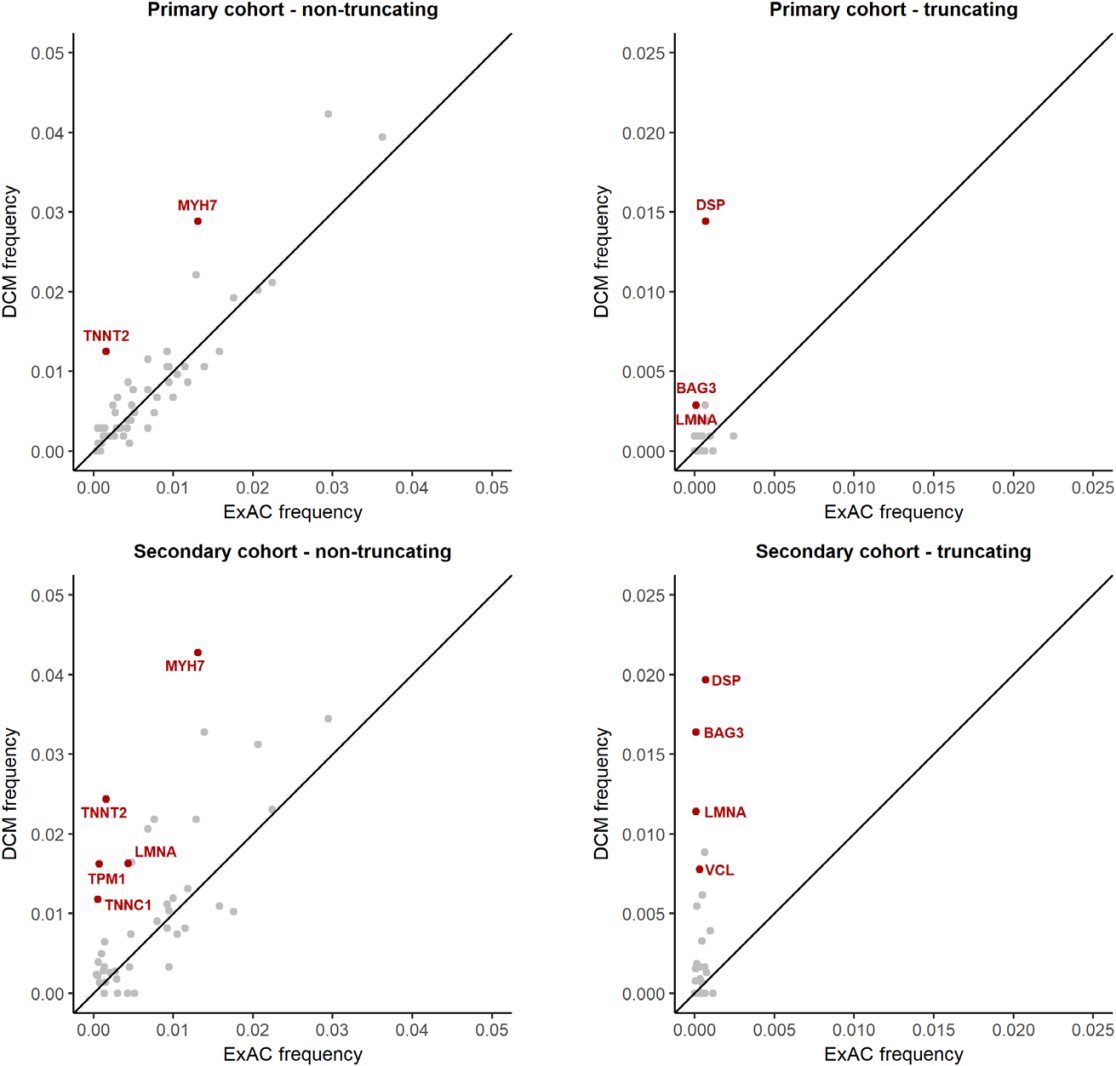
**Comparison of the prevalence of rare protein-altering variation between dilated cardiomyopathy (DCM) cases and the Exome Aggregation Consortium (ExAC) reference dataset.** Genes with a statistically significant association with disease are highlighted in red. Results are shown separately for truncating and nontruncating variants, and for primary and secondary cohorts. Truncating variants in *TTN*, robustly associated with DCM (prevalence 11.3% in primary DCM cohort and 11.7% in secondary cohort, vs 0.55% ExAC frequency), are not shown on these axes.

Protein-truncating variants in constitutive exons (Percent Spliced In>90%) of *TTN* and in *DSP* were significantly enriched in patients with DCM compared with healthy controls (*TTN* 11.3% DCM vs 0.4% healthy controls, *P*=6.2×10^−27^; *DSP* 1.4% vs 0.0%, *P*=4.2×10^−3^). We confirmed these findings using ExAC as a control cohort, and with this increased power were additionally able to detect disease associations with truncating variants in *BAG3* (0.3% DCM vs 0.007% ExAC, *P*=9.1×10^−3^) and *LMNA* (0.3% vs 0.007%, *P*=1.0×10^−2^), as well as nontruncating variants in *MYH7* (2.9% vs 1.3%, *P*=4.9×10^–3^) and *TNNT2* (1.3% vs 0.2%, *P*=2.2×10^–6^; Table; Figure 3; details of single gene burden tests in Table IX in the online-only Data Supplement and single variants identified in primary cohorts in Tables III and IV in the online-only Data Supplement).

**Table.**
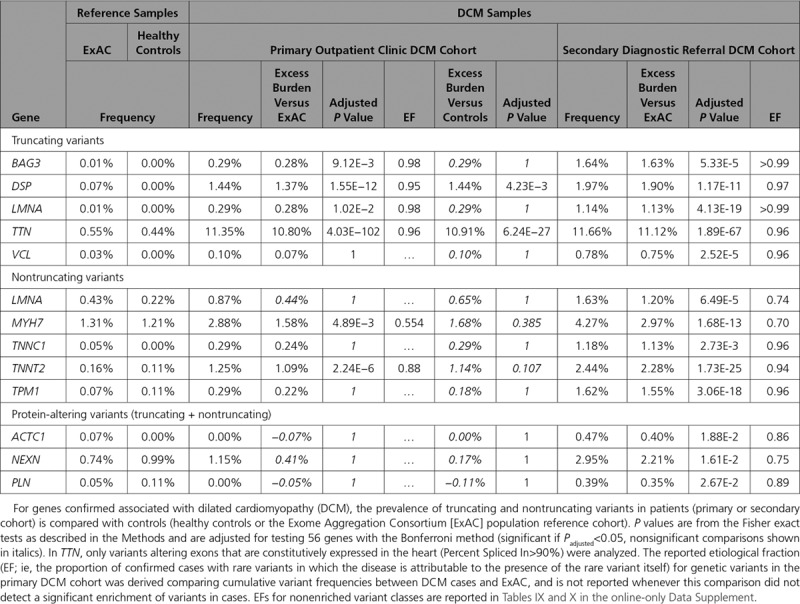
Genes With Significant Rare Variant Enrichment in DCM

#### DCM Secondary Diagnostic Laboratory Referral Cohort

The secondary referral DCM cohort provides an opportunity for replication, and also addresses potential biases affecting our primary DCM cohort caused by the CMR-based recruitment strategy and the underrepresentation of pediatric patients. Ten of the analyzed genes were not sequenced in the secondary cohort (*ACTA1, DNAJC19, FKTN, NKX2-5, NPPA, SGCB, TBX20, TBX5, TMPO*, and *ZBTB17*), and the number of cases sequenced per gene ranged from 135 to 1498. Comparison of the diagnostic referral cohort with ExAC replicated all significant associations observed with the primary outpatient cohort and additionally showed truncating variants in *VCL* (0.8% vs 0.03%, *P*=2.5×10^−5^) and nontruncating variants in *TPM1* (1.6% vs 0.07%, *P*=3.1×10^−18^), *LMNA* (1.6% vs 0.4%, *P*=6.5×10^−5^), and *TNNC1* (1.2% vs 0.5%, *P*=2.7×10^−3^) to be significantly enriched in DCM (Table; Figure 3; details of single gene burden tests in Table X in the online-only Data Supplement and single variants identified in the secondary DCM cohort in Tables V, VI, and VII in the online-only Data Supplement).

In addition, although no significant enrichment was observed for either truncating or nontruncating variants (analyzed separately) in *ACTC1, NEXN*, and *PLN*, patients with DCM of the secondary diagnostic referral cohort were significantly enriched for aggregated protein-altering variants in these 3 genes (truncating + nontruncating; *ACTC1* 0.47% vs 0.07%, *P*=1.9×10^−2^, *NEXN* 2.9% vs 0.7%, *P*=1.6×10^−2^, *PLN* 0.4% vs 0.04%, *P*=2.7×10^−2^).

We found variants in *TPM1* and *VCL* to occur significantly more often than expected in pediatric DCM (*TPM1*: 10 of 12 carriers for whom age of onset was known were <18 years of age, FDR-adjusted exact binomial test *P*=2.7×10^−2^, *VCL*: 5 of 5 <18 years, *P*=4.3×10^−2^; details in Notes in the online-only Data Supplement).

#### Integrating Findings Across Cohorts

**Figure 4.**
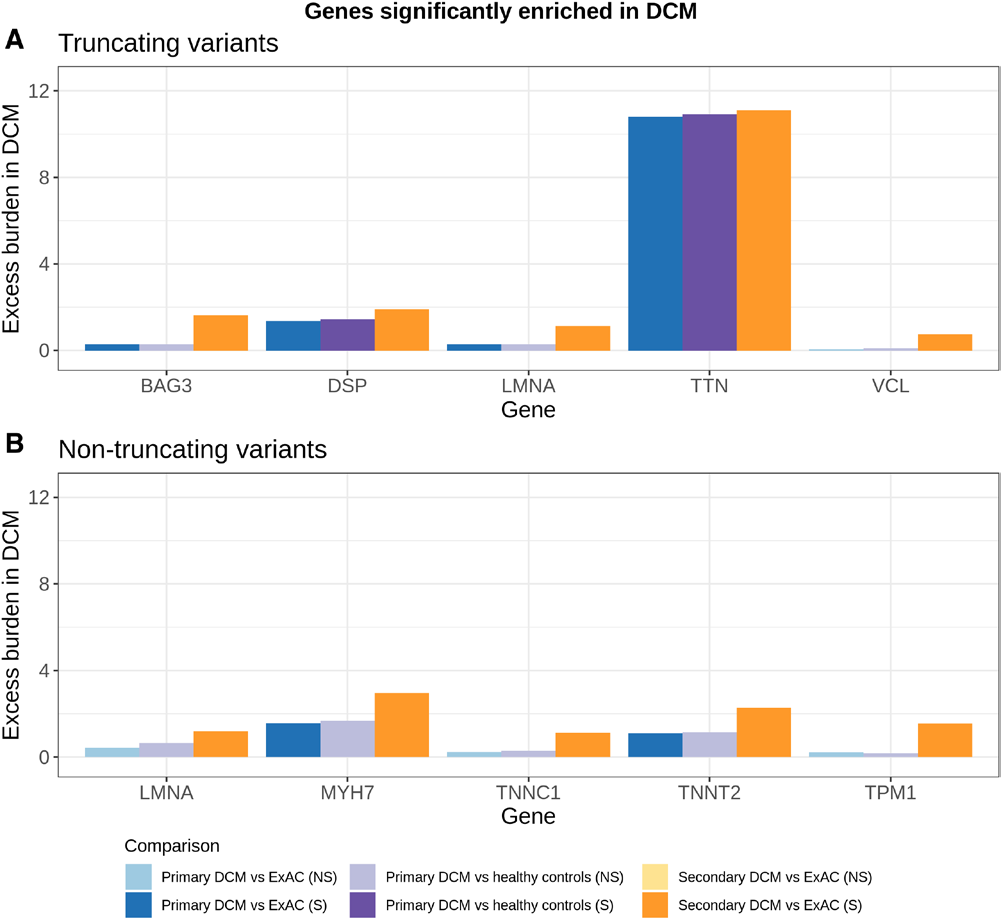
**Case excess of rare variants in genes significantly enriched in dilated cardiomyopathy (DCM).** The excess burden of rare protein-altering variation (above the population background rate) provides an estimate of the proportion of cases attributable to variation in each gene under a dominant monogenic inheritance model. Genes with a significant enrichment of rare truncating (**A**) and nontruncating (**B**) variants in patients with DCM compared with controls are shown (with the exceptions of *ACTC1*, *PLN*, and *NEXN*, where we detected an enrichment of joint truncating and nontruncating variation in DCM but not when the 2 variant classes were analyzed separately). Results are summarized for 3 comparisons as shown in Figure 2. Statistically significant enrichments are shown in saturated hues. ExAC indicates Exome Aggregation Consortium; NS, not significant; and S, significant.

In summary, DCM was associated with truncating variants in *TTN* and *DSP* in all comparisons. A significant association with truncating variants in *BAG3* and *LMNA*, and nontruncating variants in *MYH7* and *TNNT2*, was demonstrated when comparing each DCM cohort to ExAC. The enrichment of truncating variants in *VCL*, and nontruncating variants in *TPM1*, *LMNA*, and *TNNC1*, was unique to the diagnostic referral cohort (Table; Figure 4), as was the case for aggregate truncating and nontruncating variation in *ACTC1*, *NEXN*, and *PLN*. The total excess burden of rare variants (in these 13 significantly enriched variant classes), which provides an estimate of “diagnostic yield” for testing these genes, was 16.7% (95% CI, 14.2%–19.2%) in the unselected primary cohort and 25.9% (95% CI, 23.0%–28.9%) in the diagnostic referral cohort.

The total excess burden of rare variation in each gene provides an estimate of the eventual diagnostic yield that might be achieved with optimum variant interpretation. Of note, *TTN* accounts for ~11% of DCM cases in these cohorts, whereas each other gene contributes ~0.3% to 3% (Figure 4). Detailed results of all comparisons are provided in Tables IX and X in the online-only Data Supplement.

#### Interpretability of Variant Classes Measured by Etiological Fraction

**Figure 5.**
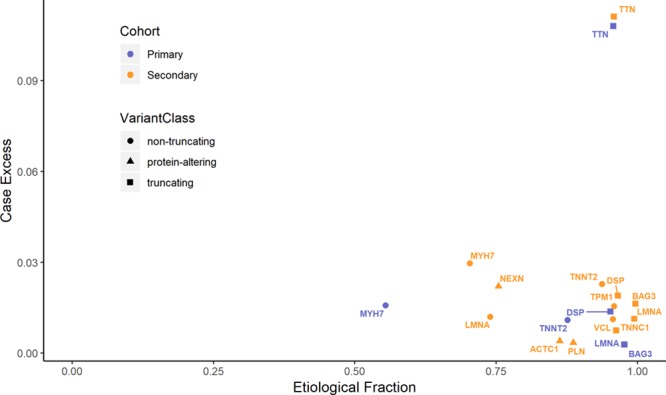
**Known and interpretable classes of genetic variants in dilated cardiomyopathy (DCM).** The case excess (the difference in rare variant prevalence between cases and controls) provides an estimate of the contribution of a gene/variant class to DCM (proportion of cases explained by that gene/variant class) and is plotted against the etiological fraction, a measure of the interpretability of rare variants detected in patients (the proportion of cases with a variant where that variant is likely causative). Truncating and nontruncating variants are shown separately for each DCM cohort.

The etiological fraction estimates the prior probability that a rare variant of a particular variant class is pathogenic if found in a patient with disease. As expected, we observed truncating variants in the genes with a confirmed disease association to have high etiological fractions, reflecting the rarity of such variants in the population, whereas etiological fractions for nontruncating variants were more modest (eg, 0.99 and 0.74 for truncating and nontruncating variants in *LMNA*, respectively (Figure 5; Table). In contrast, etiological fractions for nonenriched genes were low and nonsignificant (Tables IX and X in the online-only Data Supplement), indicating that the prior probability that a novel variant in these genes is pathogenic is low, and a great deal of evidence would be required to assert pathogenicity. Of note, the likelihood of pathogenicity for a rare variant found in a patient as estimated by etiological fraction (interpretability) is distinct from, but not independent of, both the probability that it will inform treatment, and the potential to predict disease onset in relatives (penetrance). For genes like *MYH7* or *LMNA*, known pathogenic variants are highly penetrant in families and informative for clinical care, despite both genes having a relatively low etiological fraction for nontruncating variants in aggregate, because of substantial levels of rare benign variation in the population. In contrast, truncating variants in *TTN* are highly likely to be disease-causing when found in an individual with DCM but have been shown to have reduced penetrance in the wider population.

#### Posterior Quality Control on Ethnicity Stratification

The per-gene excess of rare variants in primary patients with DCM (N=1040) over the full ExAC dataset and the one observed using only samples of European descent (white primary DCM cases (N=721) versus the Non-Finnish European subset of ExAC (N=33 270) showed strong positive correlation (R^2^ = 0.975, Figure VI in the online-only Data Supplement). None of the 44 genes not enriched for rare variation in DCM were associated with the condition in the 721 white patients (*P* values in the range 0.39–1), and the prevalence of the 13 enriched variant classes was comparable in ExAC vs the Non-Finnish European subset of ExAC as well as in primary DCM vs primary white patients with DCM (*P* values in the range 0.15–1). In addition, we recently showed that population stratification was not a significant confounding factor in the subset of the secondary clinical DCM cohort included in our previous analysis.^4^ These findings suggest that the results of this analysis are robust to population stratification.

### Genotype in Relation to Age and Family History

Age at diagnosis was significantly different between genotype-positive and genotype-negative patients with DCM (mean±SD, 47.8±16.2 years vs 56.1±14.3 years, respectively, *P*=4.5×10^–10^), but we did not detect a significant effect of specific gene/variant classes (*P*=0.3). Patients with positive family history are diagnosed earlier, both within genotype-positives (41.5±16.3 years vs 49.1±15.6, *P*=0.007) and within genotype-negatives (50.1±16.3 years vs 56.4±14.1, *P*=0.02), and are characterized by a higher yield of potentially pathogenic variants (40.0% vs 17.6%, *P*=1.4×10^−6^). Full details are provided in Table XI in the online-only Data Supplement.

## Discussion

In this study, we have compared the burden of rare protein-altering variation in DCM-associated genes in up to 2538 patients with DCM—to our knowledge the largest such study to date—to the background genetic variation in both healthy controls and the ExAC reference population.

We propose that the variant classes significantly enriched in 1 or both DCM cohorts are likely to be most relevant for genetic testing in DCM. These were truncating variants in *TTN*, *DSP*, *LMNA*, *BAG3*, and *VCL*, and nontruncating variants in *MYH7, TNNT2, TNNC1, LMNA*, and *TPM1* (Table). Analyzing truncating and nontruncating variants jointly, variants in *ACTC1*, *NEXN*, and *PLN* were also associated with DCM. *BAG3* is one of the more recently implicated DCM genes and was not assessed in our previous study. Here, a significant excess of *BAG3*-truncating (but not nontruncating) variants was observed in DCM, consistent with multiple reports of the cosegregation of *BAG3*-truncating alleles with DCM in large pedigrees (Table XII in the online-only Data Supplement).

The analysis of 2 distinct patient cohorts represents a strength of the study, as it enables the dissection of the genetic basis of DCM in different contexts. Our primary DCM cohort evaluates the contribution of known genetic determinants to DCM across a broad range of cases, including less severe, later-onset and nonfamilial cases that are likely underrepresented in cohorts selectively referred for diagnostic sequencing, because genetic testing has primarily been recommended for patients with strong family history and/or conduction disease.^12^ It also enables us to compare data from cases and controls with standardized sequencing and uniform data processing to minimize technical artefacts. However, although CMR evaluation in this cohort provides accurate deep phenotyping, this may also introduce bias, eg, against infant patients or those with implanted cardiac devices. This is likely to impact genes associated with pediatric forms of DCM,^13^ and genes, such as *LMNA*, that are associated with DCM in combination with conduction disease and arrhythmia likely to require device implantation. Consistent with this, rare *LMNA* variants were >2.5-fold more frequent in the diagnostic referral cohort (2.8%, in line with other reported non-CMR cohorts) compared with the CMR-characterized primary outpatient cohort (1.1%), likely reflecting a combination of enrichment in the diagnostic referrals (increased likelihood of referral in the presence of conduction disease), and depletion in the primary cohort (less likely to undergo CMR after device therapy).

The genetic determinants of pediatric DCM may be distinct from adult-onset DCM,^13,14^ and our cohorts differ in their representation of childhood-onset disease (primary patient cohort predominantly adult-onset (848 of 863 [98.3%] older than 18 years); diagnostic referral series containing a substantial pediatric population (Figure I in the online-only Data Supplement, 286 of 691 [40.4%] patients with age information available younger than 18 years). One explanation for associations only observed in the diagnostic referral cohort may be a particular association with early-onset disease. Among variant classes enriched in the diagnostic referral cohort, we found that variants in *TPM1* and *VCL* were significantly more prevalent in patients below 18 years of age compared with older patients (*P*=2.7×10^–2^ and *P*=4.3×10^−2^, respectively). This supports previous reports describing *TPM1* as an important early-onset DCM gene.^15,16^ Of note, protein-altering variants in *NEXN* and in *ACTC1* were also carried primarily by pediatric patients (*NEXN*, 6 of 7 and *ACTC1*, 5 of 6 carriers), although the prevalence was not significantly different in adults vs children after correcting for multiple testing.

We observe a considerable difference in diagnostic yield between cohorts (16.7% in primary, unselected cohort vs 25.9% in secondary diagnostic referral cohort), concordant with selective referral for genetic testing of patients with clinical features predictive of a monogenic etiology, reflecting clinical practice guidelines during this period. Some published estimates of potential diagnostic yield, including our previous study,^4^ focused only on variants with a clinical laboratory classification of pathogenic or likely pathogenic, which will provide a very conservative estimate. The excess of rare variants in cases over controls represents what could be attainable with improved variant discrimination.

The significant enrichment of rare variation in disease cases constitutes strong evidence in favor of pathogenicity. Forty-four genes analyzed showed no excess of rare variation in DCM. Although this does not definitively rule out a role in disease, it implies that variants in these genes will most likely not be interpretable in the diagnostic context, unless strong additional evidence for pathogenicity of the variant in question is collected or previously published, and the proportion of cases with monogenic dominant DCM attributable to these genes will be small. Notably, previous work in hypertrophic cardiomyopathy found that most genes without a demonstrable excess of rare variation in cases had been implicated through candidate-gene approaches with limited, if any, statistically robust human genetic evidence for their involvement in disease,^5^ findings recently confirmed by the ClinGen hypertrophic cardiomyopathy curation study.^17^ Similarly, all but one of the genes implicated in Brugada syndrome have now been refuted by ClinGen curation,^18^ in concordance with findings from burden testing.^19^ Examination of the original reports for selected genes proposed as causative for DCM are likely to show that many did not fully account for population variation or formally test for statistical association, and often did not provide either robust segregation evidence or convincing functional data.

Nonetheless, some of the genes without demonstrable association here may still prove to be causative of DCM. Power to demonstrate association will be limited for genes causing only a small proportion of DCM (eg, evidence of cosegregation of nontruncating variants in *SCN5A* with arrhythmogenic DCM is reported alongside functional data demonstrating altered sodium channel function^20^), or for genes in which variation in specific functionally important residues or genic subregions is disease-associated, but not detectable against background variation in other regions tolerant of variation. For example, the *TTN* missense variant p.Trp930Arg shows convincing cosegregation with DCM,^21^ supported by functional data,^22^ yet nontruncating variants in *TTN* are not significantly enriched in any of the comparisons we performed (~29%–30% in DCM vs ~27.5% in controls), with a very high background variation rate in the population. Most missense variation in *TTN* is diagnostically uninterpretable, representing an urgent need for strategies to discriminate *TTN* nontruncating variants that contribute to DCM. This demonstrates the need for systematic evaluation efforts—such as the ClinGen initiative—that review all lines of human genetic evidence alongside functional studies.

These findings will inform genetic testing for DCM, both in the composition of test gene panels and the interpretation of results. Restricting clinical testing to genes validated for the disease in question, and specific functionally relevant variant classes therein (in combination with stringent variant interpretation), is of critical importance, to avoid false-positive results and a proliferation of variants of uncertain significance from large gene panels. Both cause significant harm, through anxiety and wasted resources. For variant interpretation, etiological fraction estimates from case-control studies can be directly useful, estimating the prior probability of pathogenicity for different variant types, and determining the level of additional evidence required to render a variant actionable for family management. These values can also be directly incorporated into the variant classification framework as we have previously demonstrated,^23^ leading to a significant increase in sensitivity of genetic testing.

### Limitations

The TruSight Cardio panel was designed in 2015 and does not include genes implicated in DCM more recently, such as *FLNC.*^24^ In addition, *RBM20* was excluded from analysis because of suboptimal sequencing coverage in ExAC. Convincing evidence for DCM pathogenicity has been published for specific variants in this gene,^25,26^ but further studies are needed to evaluate to what extent novel *RBM20* variants are interpretable in a diagnostic context.

There are limitations inherent in the use of ExAC as a reference population dataset for comparison with a genetic disease like DCM, including imperfect sequencing coverage from whole-exome sequencing assays, the unknown prevalence of DCM among individuals in ExAC, and the use of ExAC to define rare variants and as a control cohort that could introduce biases. However, our sequencing quality control based on presumably benign synonymous variants and the adjustment for variable sequencing coverage in ExAC enabled calibration of burden testing results. This is demonstrated by the comparability of ExAC with the 912 confirmed healthy controls over the frequency of rare protein-altering variants, the comparable detection rate of synonymous variants in ExAC and in our panel sequencing data as well as by the unbiased distribution of *P* values for single-gene comparisons of synonymous variants burden (Figures III, IV, and V in the online-only Data Supplement).

This evidence cumulatively suggests that ExAC—with appropriate quality control—can be reasonably used as control population. In our study, these analyses are complementary to a well-controlled comparison of uniformly sequenced and processed cases and controls. Any residual bias arising from comparisons of panel-sequenced cases with ExAC would be expected to yield false-positive associations, rather than false-negative, as TruSight Cardio yields more complete target coverage and greater sequencing depth than whole-exome sequencing in ExAC. As the primary finding of this study is that most implicated genes lack any enrichment in cases, our results are robust to the most likely direction of bias.

Our analyses in this study focused on variation in protein-coding regions, as the technology adopted does not fully characterize other variant classes such as noncoding, epigenetic, and large structural variants, although we consider them unlikely to underlie a substantial burden of Mendelian disease given the lack of enrichment observed for protein-altering variants in most genes studied here. Future studies based on whole-genome sequencing of patients with DCM will address these issues, and eliminate any bias associated with incomplete coverage of the coding region in targeted and exome sequencing due, for example, to GC-rich regions (ie, sequence regions with high content of guanine and cytosine).^27^

Survival bias cannot be excluded, so that cohorts may be depleted of variants causing severe early-onset DCM, and some healthy controls may develop DCM in the future. Neither of these potential biases should have a large effect on our results: given the prevalence of DCM (1 in 250), we would expect <5 healthy controls to potentially develop DCM in the future, and the inclusion of the secondary diagnostic laboratory cohort—enriched in pediatric patients—allows us to assess early-onset DCM in younger patients. Because of consent and data privacy issues, we were unable to assess for significant differences in characteristics of patients who did not agree to participate in this study, but we believe this is unlikely to influence generalizability of our findings.

### Conclusions

We have comprehensively evaluated rare protein-altering variants in genes implicated in DCM in a large cohort, and identified 12 genes that have robust evidence of disease association. We estimated the contribution of these genes to disease, which indicates the potential diagnostic yield of sequencing and the prior probability that a novel uncharacterized variant identified in an individual with disease is pathogenic, and is informative as to clinical interpretability of variation in this gene. These genes have clear diagnostic utility in a clinical setting.

## Sources of Funding

This work was supported by the Wellcome Trust (107469/Z/15/Z), the Wellcome Transforming Genomic Medicine Initiative (200990/A/16/Z), the British Heart Foundation (SP/10/10/28431 and RG/19/6/34387), the Medical Research Council (MR/M003191/1), the National Institute for Health Research Cardiovascular Biomedical Research Unit based at Royal Brompton and Harefield National Health Service Foundation Trust and Imperial College London, the National Institute for Health Research Biomedical Research Centre based at Imperial College London Healthcare National Health Service Trust and Imperial College London, the Academy of Medical Sciences (SGL015/1006), the Fondation Leducq (11 CVD-01), a Health Innovation Challenge Fund award from the Wellcome Trust and Department of Health, United Kingdom (HICF-R6-373), the Italian Ministry of Health (RF-2013-02356787 and NET-2011-02347173), and Regione Toscana (Tuscany Registry of Sudden Cardiac Death [ToRSADE - FAS Salute 2014]). Dr Mazzarotto is supported by a postdoctoral research fellowship from the University of Florence. Dr Walsh is supported by an Amsterdam Cardiovascular Sciences fellowship. Dr Whiffin is supported by a Rosetrees and Stoneygate Imperial College Research Fellowship. This publication includes independent research commissioned by the Health Innovation Challenge Fund, a parallel funding partnership between the Department of Health and the Wellcome Trust. The views expressed in this work are those of the authors and not necessarily those of the funders.

## Disclosures

None.

## Supplementary Material


