# Supplementary material for: Reevaluating the Genetic Contribution of Monogenic Dilated Cardiomyopathy
Source: Circulation. 2020 Jan 27;141(5):387–98. doi: 10.1161/CIRCULATIONAHA.119.037661 (PMC7004454; doi:10.1161/CIRCULATIONAHA.119.037661)
Supplement: Supplementary file 1 [file cir-141-387-s001.pdf]

SUPPLEMENTAL MATERIAL

SUPPLEMENTAL METHODS

Research DCM and control cohorts

The primary outpatient DCM cohort comprised 863 patients recruited to the NIHR Biobank at the Royal Brompton Hospital, London and 177 to the Singapore Biobank at the National Heart Centre Singapore. Patients from the London cohort consisted of consecutive referrals to the imaging unit from the dedicated cardiomyopathy service at Royal Brompton Hospital, London, and a network of 30 regional hospitals. Patients were referred for diagnostic evaluation, family screening, or assessment of DCM severity.

For patients with cardiovascular magnetic resonance (CMR), DCM was diagnosed based on evidence of left ventricular dilation and systolic impairment with reference to age, gender and body surface area adjusted nomograms[1]. For patients with echocardiography, DCM was diagnosed in the presence of left-ventricular end-diastolic diameter >117% of that predicted for age and body surface, and left-ventricular ejection fraction <45% and/or fractional shortening <25%[2], in the absence of known coronary artery disease (presence of subendocardial LGE suggestive of previous myocardial infarction, >50% stenosis in 1 or more major epicardial coronary arteries, or need for previous percutaneous coronary intervention or coronary artery bypass grafting), abnormal loading conditions (uncontrolled hypertension or significant primary valvular disease), or toxin exposure (alcohol consumption in excess of 80 g/day for 5 years meeting criteria for alcoholic cardiomyopathy). Participants who had known cardiovascular or metabolic disease were excluded from the healthy volunteers prior to CMR scanning. Subjects taking prescription medicines were also excluded but simple analgesics, antihistamines, and oral contraceptives were acceptable. Female subjects were excluded if they were pregnant or breastfeeding. Standard safety contraindications to magnetic resonance imaging were applied including a weight limit of 120 kg.

Targeted sequencing and bioinformatics data processing

All patients and controls in the primary research cohorts underwent targeted sequencing of 174 genes associated with inherited cardiac conditions using the custom developed Illumina TruSight Cardio Sequencing Kit for sequence capture. Samples were sequenced using the Illumina NextSeq 500 or the Illumina MiSeq platforms. Targeted DNA libraries of 48 (NextSeq 500) or 12 (MiSeq) samples were prepared using the TruSight Cardio kit with sample and library quality control performed using the Qubit 3.0 fluorometer (Life Technologies) and the TapeStation 2200 electrophoresis system (Agilent Technologies). Libraries were sequenced as 150 bp paired-end reads. Demultiplexing of sequence data was performed using NextSeq Control software or Bcl2FastQ conversion 2.16[3] and resulting FastQ files subjected to quality control with the FastQC[4] v.0.10.14. Low quality reads (Q<20, window_size 5) were trimmed using PrinSeq[5] v0.20.4, and sequences aligned to the GRCh37/hg19 reference genome using BWA[6] v0.7.10. Picard[7] v1.115 and GATK[8] v3.2-2 were used to mark duplicate reads and perform local realignment around indels and base quality score recalibration. Fifty-four genes (of the 56 analysed) were characterised by optimal coverage in our 1952 samples sequenced on the TruSight Cardio panel, with a mean of >99% of bases covered at ≥10x sequencing depth for each gene. Two genes had marginally reduced coverage: *NKX2.5* (98.8%) and *LDB3* (98.3%), yielding an overall per-gene mean coverage of 99.8±0.3%. Variant calling was performed using GATK Unified Genotyper and Haplotype caller on each sample separately, and variants called by either caller were included.

Sequence data from patients of the secondary clinical DCM cohorts was generated using a range of mutation scanning and direct sequencing techniques of varying sensitivity (High-resolution DNA melting, WAVE dHPLC, LightScanner®, DNA microarray [Cardiochip], Sanger sequencing, targeted next-generation sequencing). These targeted tests are designed to cover the coding regions and splice sites of the genes of interest; the analytical sensitivity of these methods is estimated to be in the region of 98-100% (data from in house validation). As all putative pathogenic variants are confirmed by Sanger sequencing the rate of false positive variant calls will be negligible.

Variant quality-based filtering and quality control

Variant calls in isolated samples from primary cohorts that did not pass all GATK quality filters and/or with a quality-by-depth (QD)<4 and/or read depth <10x and/or an allelic balance <0.2 in were not included in our counts due to the high likelihood of being false positives.

Variant sites covered at less than 10x in <95% samples and/or that did not pass all quality filters in ExAC were masked out from all cohorts from the analysis given the consequent unreliability of frequency measures for variant alleles at such sites.

After masking out from analysis such positions, a bespoke algorithm developed on the basis of the framework proposed by Guo et al.[9] was adopted to calibrate additional variant quality cut-offs based on the variant site-specific QD and VQSLOD values in ExAC. The algorithm performs an iterative optimization of the genomic inflation factor ($GIF_{adjusted}$, calculated with the adjusted formula proposed by Guo et al.) by:

- testing 3600 different percentile-based cut-off combinations of QD and VQSLOD between the 0.5^{th} percentile and the 30th percentile.
- amongst the various combinations yielding an optimal $GIF_{adjusted}$ (0.95 < GIF < 1.05, indicative of the absence of systematic bias), prioritizing the combination that minimizes the number of variant sites masked out from analysis.
- comparing the resulting burden of rare synonymous variants between the targeted panel data (n=1952 samples, 1040 primary DCM cases + 912 primary healthy controls) vs ExAC both at the single gene level and at the gene-set (n=56 genes) level, ensuring the absence of significant burden differences.

As a result of this, the optimal calibration was found masking out from analysis variants at positions with QD < 10.8622 (14.5^{th} percentile) and/or VQSLOD < -1.041068 (9th percentile). The resulting adapted $GIF_{adjusted}$ was 1.008 (Figure S3), single-gene burden testing p-values ranged between 0.65 and 1 (Table S8 and Figure S4) and the frequency of rare synonymous variants was comparable at the gene-set level (31.6% in panel data vs 30.3% in ExAC, p=0.25). The same absolute QD and VQSLOD cut-offs (10.8622 and -1.041068, respectively) were

applied on subsequent tests on protein-altering variants. We have also excluded from analysis variants carried by patients of the secondary diagnostic laboratory cohorts below these cut-offs in ExAC. Although we acknowledge that cut-offs were derived using data from the primary cohorts, we chose this strategy for increased consistency, and in order to use equal per-gene ExAC variant counts in all comparisons.

Burden testing

Throughout this work, we used 0.0001 as frequency cut-off to define rarity, and to exclude variants that are not plausibly pathogenic protein-altering variants under a dominant model. After the onset of this work, a more stringent and variant-specific allele frequency adjustment framework has been proposed[3] – with 8.4×10^{-5} estimated as maximum credible allele frequency for any DCM-causing variant. The threshold used here is very close to this value and only slightly more conservative, and was retained for backwards compatibility with prior analyses. The central findings are not changed by adoption of the frequency cut-off calculated with the more recent framework (data not shown), given the very small amount of filtered variants with $8.4 \times 10^{-5} < MAF < 0.0001$.

Statistics

Power calculations were performed using the functions "pwr.2p2n.test()" and "ES.h" of the R package "pwr"[10] providing sample sizes and specifying a target power of 80%, probability of type I error of 5% and the option "alternative=greater" since we tested protein-altering variation burdens for enrichment in cases rather than bi-directional differences.

Confidence intervals for aggregate estimations of proportion of cases explained by the aggregate enriched variant classes in primary and secondary DCM cohorts were calculated with the built-in "prop.test()" R function[11]. In specifying variant frequencies for ExAC and the secondary DCM cohort (characterized by a different number of sequenced individuals over each gene), the

average number of individuals sequenced over the enriched genes was used as total number of individuals.

Confidence intervals for etiological fractions (reported in Supplementary Tables S9-S10) have been calculated using the method developed by Hildebrandt et al.[12].

Exact binomial tests were performed using the built-in function “binom.test()” in R[11], specifying “alternative=’greater’” to perform one-tailed tests to assess enrichment of pediatric cases.

LIST OF SUPPLEMENTAL TABLES (in .xlsx file)

Supplemental Table 1 — Baseline demographic characteristics and cardiometabolic risk factors of the DCM probands and healthy volunteers from the primary cohorts. Data for DCM patients are reported for the subset of patients for whom information were available (demographics for the 863 probands recruited in London, cardiac phenotype for 752 of them). Counts and relative percentages are reported for categorical variables, while continuous variables are described by mean±standard deviation.

Supplemental Table 2 — Full list of the genes with ≥1 variant reported pathogenic for DCM between 1996 and 2015 in the HGMD (professional version 2015.3) and targeted by the Illumina Trusight Cardio panel alongside transcript details, number of cases and controls sequenced per cohort, and first publication associating each gene with DCM. *The RBM20 ExAC denominator includes non-QCed variants (as RBM20 was not included in our main analysis).

Supplemental Table 3 — Full list of rare variants (protein-altering and synonymous) carried by the in-house DCM patients of the primary research cohort, sequenced with the Illumina Trusight Cardio panel.

Supplemental Table 4 — Full list of rare variants (protein-altering and synonymous) carried by the in-house healthy controls of the primary research cohort, sequenced with the Illumina Trusight Cardio panel.

Supplemental Table 5 — Full list of rare variants carried by the previously published (Walsh et al, Genetics in Medicine, 2015)[13] diagnostic referral DCM cohort from the Oxford Medical Genetics Laboratory.

Supplemental Table 6 — Full list of rare variants carried by the previously published (Pugh et al, Genetics in Medicine, 2014 and Walsh et al, Genetics in Medicine, 2015)[13,14] diagnostic referral DCM cohort from the Laboratory of Molecular Medicine.

Supplemental Table 7 — Full list of rare variants carried by the new diagnostic referral DCM cohort from the Laboratory of Molecular Medicine.

Supplemental Table 8 — Results of all per-gene comparisons between the DCM cases and HVOL controls joint primary cohorts sequenced on the Trusight Cardio panel (N=1040 DCM patients + 912 healthy controls, TOTAL=1952) and the ExAC reference population sequenced using exome sequencing for rare synonymous variants after the application of the read depth-, QD- and VQSLOD-based quality control. The resulting p-value distribution was characterized by a genomic inflation factor of 1.008. P-values are nominal (not adjusted for multiple testing).

Supplemental Table 9 — Results of all per-gene comparisons for rare (MAF<0.0001) protein-altering variant frequencies between primary research DCM samples (N=1040) and controls (both primary research healthy controls [N=912] and the ExAC reference population), including the comparison between healthy controls and ExAC performed as quality control. Reported p-values are not corrected for multiple testing. The columns named "Significant vs Controls" and "Significant vs ExAC" relate to p-values corrected for multiple testing of 56 genes (Bonferroni method). Theoretically negative values for lower bounds and values >1 for upper bounds of EF confidence intervals are capped to 0 and 1 respectively, due to the probabilistic nature of EF itself. EF=etiological fraction, OR=odds ratio, CI=95% confidence interval.

Supplemental Table 10 — Results of all per-gene comparisons for rare (MAF<0.0001) protein-altering variant frequencies between secondary diagnostic referral DCM samples (135 ≤ N ≤ 1498 sequenced samples, depending on the gene) and the ExAC reference population. Reported p-values are not corrected for multiple testing. Theoretically negative values for lower bounds and values >1 for upper bounds of EF confidence intervals are capped to 0 and 1 respectively, due to the probabilistic nature of EF itself. EF=etiological fraction, OR=odds ratio, CI=95% confidence interval.

Supplemental Table 11 — Details about significant associations (p-values reported in the main text) between genotype status (considering the 13 variant classes enriched in DCM), age at

recruitment/MRI scan and family history. Data reported in Part 1 comprise the 656 probands of the primary outpatient clinic cohort for whom age information was available and the family history status was not unknown. Data reported in part 2 comprise the 863 probands of the primary outpatient clinic cohort for whom age information was available.

Supplemental Table 12 — Summary of published evidence of the association between BAG3 variants and DCM.

SUPPLEMENTAL FIGURES AND FIGURE LEGENDS

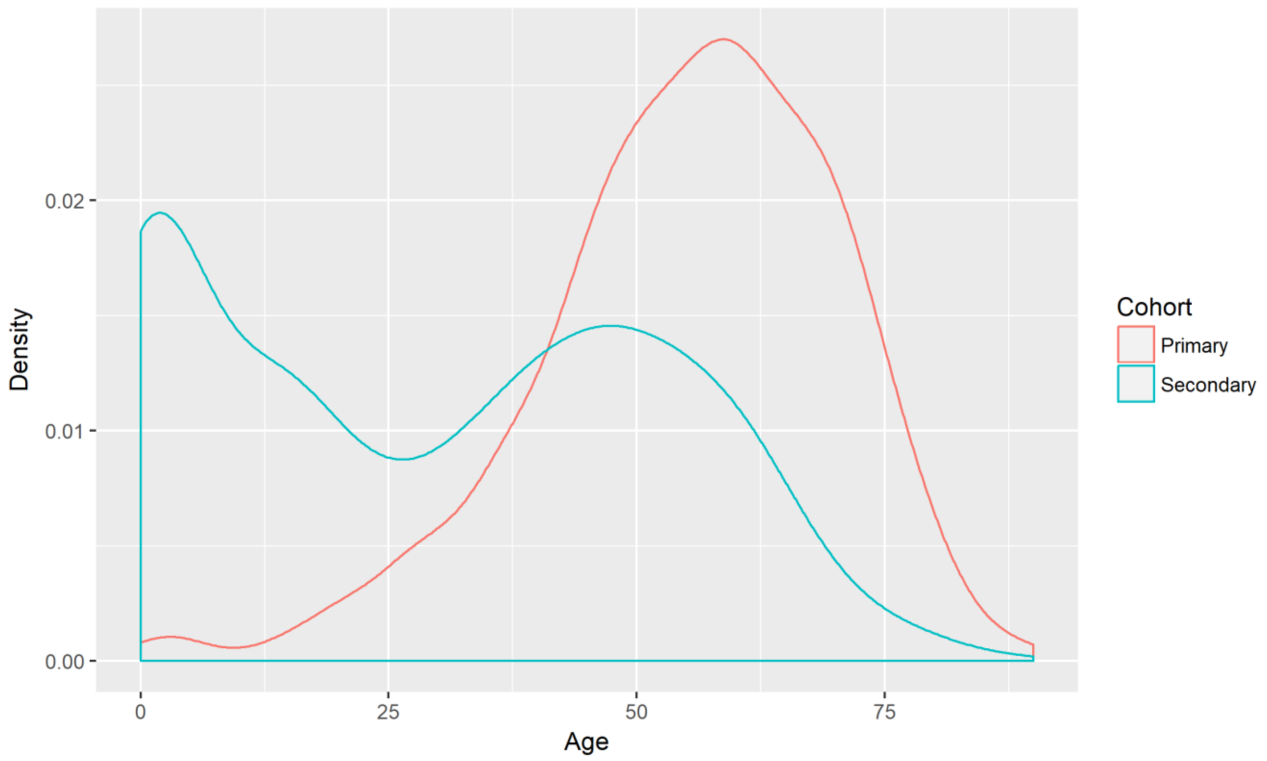

Supplemental Figure 1 — Comparison of the age distribution of DCM patients in the primary research and secondary diagnostic referral cohorts. Data available for the 863 patients from the Royal Brompton Hospital (in the research cohort) and 766 patients from the published LMM cohort (in the diagnostic referral cohort). Of note, the proportion of patients <18 years of age was 1.7% in the primary DCM cohort (15 of 863) and 41.4% in the secondary DCM patient set (286 of 691).

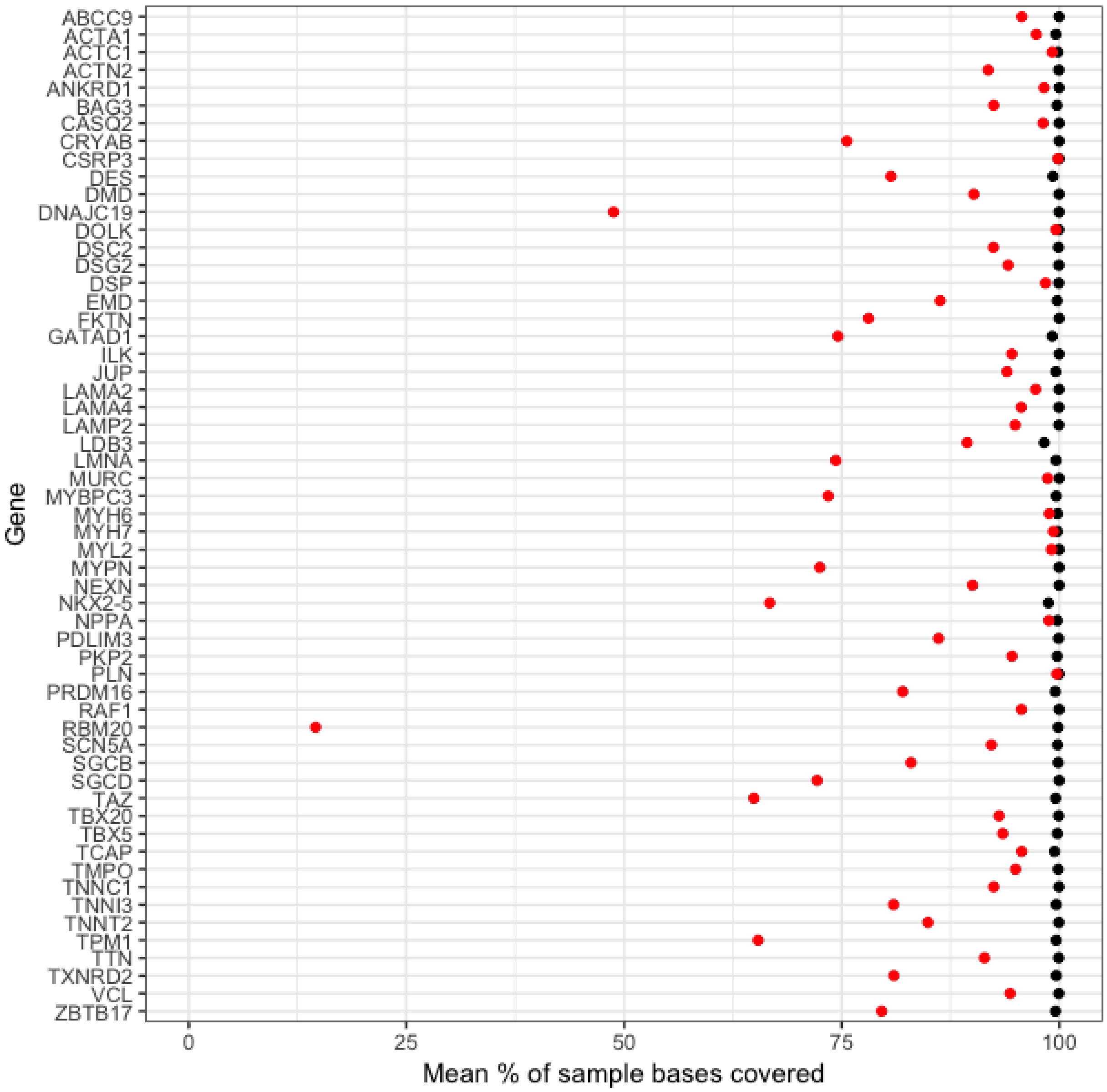

Supplemental Figure 2 — Coverage plot showing the mean percentage of sample bases covered at 10x in the targeted panel data (joint primary research DCM and healthy control cohorts [black dots]) and in the ExAC cohort (red dots) for the 57 selected genes. On the basis of these data, RBM20 (mean % of sample bases covered at ≥10x = 14.5%) was excluded from analysis, while the other 56 genes were subject to the quality control steps described in the main text and by Figures S2-S4 to ensure technical comparability between cohorts.

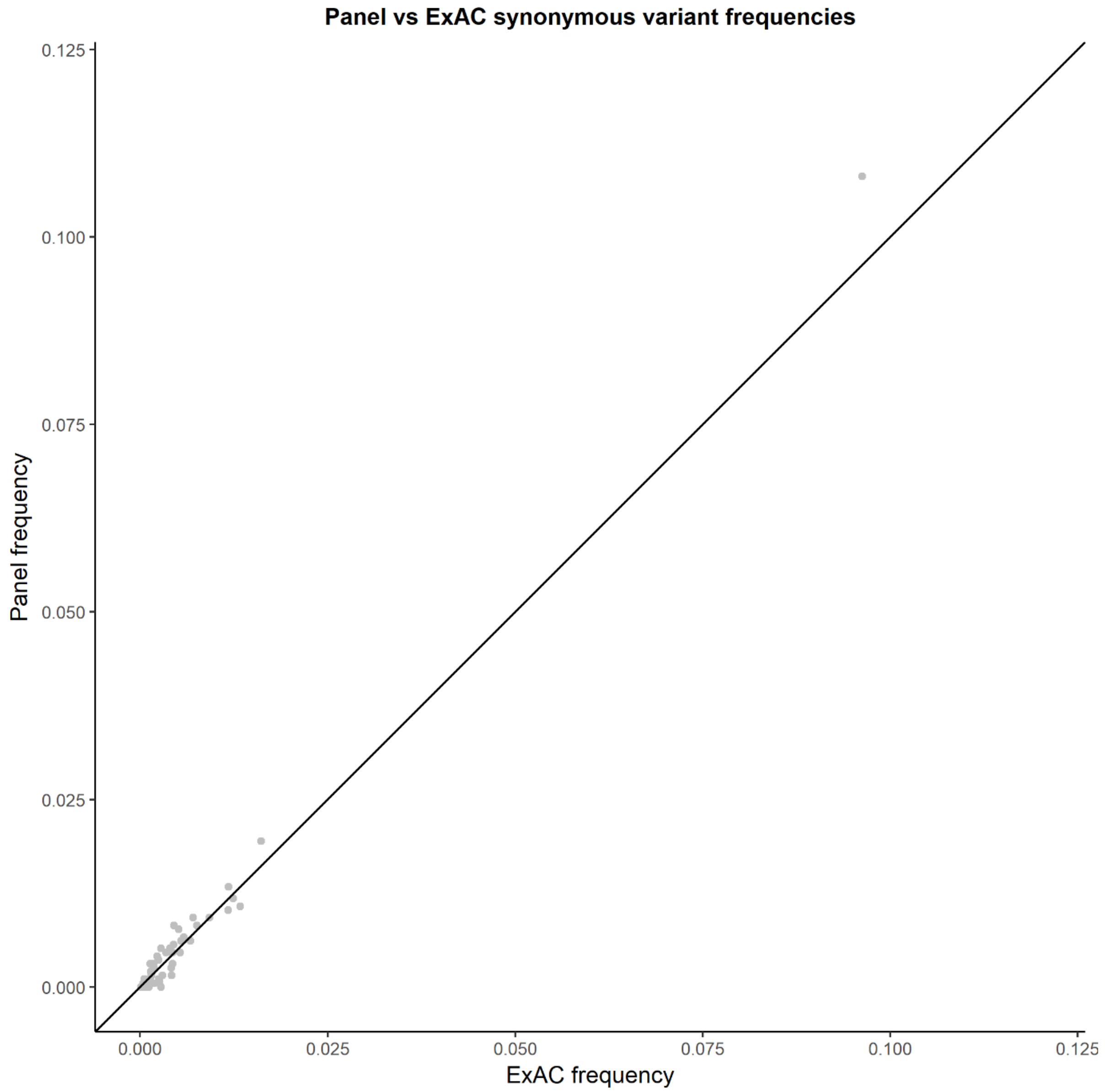

Supplemental Figure 3 — Frequency of rare (MAF<0.0001) synonymous variants in ExAC individuals compared to in-house healthy controls and DCM cases of the primary research cohort sequenced on the TruSight Cardio panel, after the application of the quality control / variant quality cut-offs combination testing. None of the genes was characterized by a significant burden difference between panel data and ExAC.

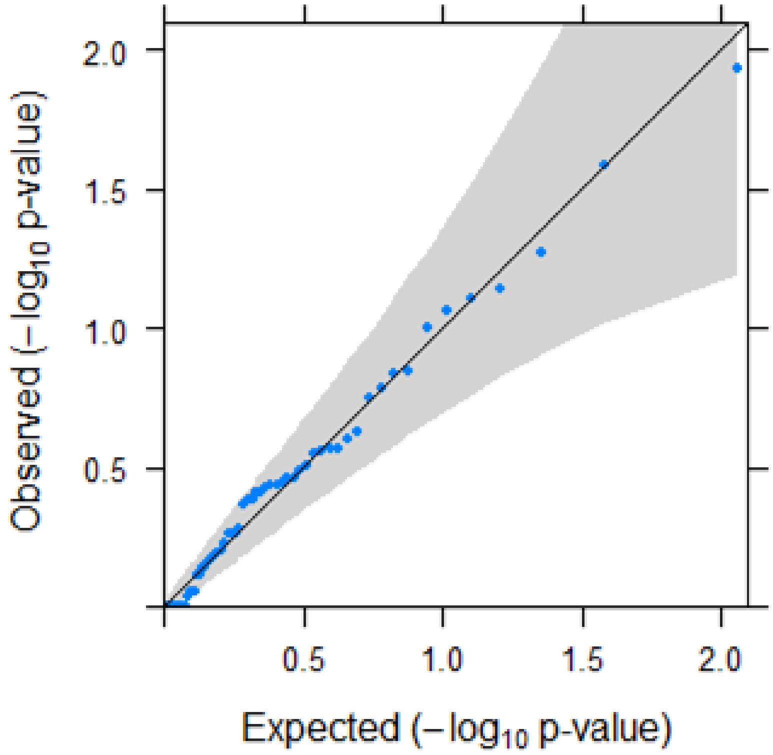

Supplemental Figure 4 — QQ plot of the expected vs observed distribution of 56 single-gene -$\log_{10}$ p-values for rare (MAF<0.0001) synonymous variants comparisons between targeted gene panel data from our primary cohorts (n=1952) and ExAC after the application of the algorithm based on the framework proposed by Guo et al.[9]. The corresponding GIF was 1.008.

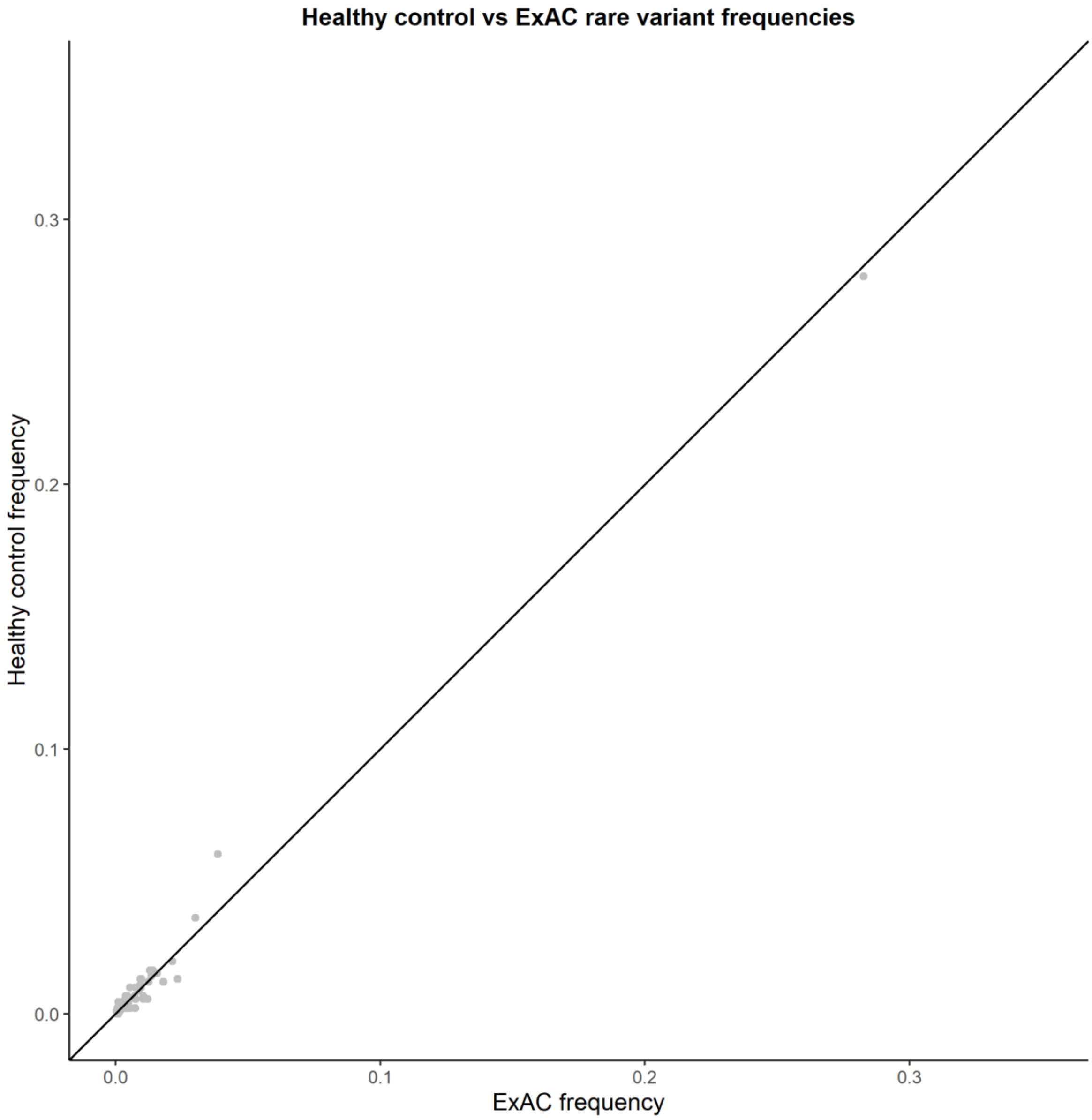

Supplemental Figure 5 — Frequency of rare (MAF<0.0001) protein-altering variants in ExAC individuals compared to in-house healthy controls of the primary research cohort. None of the genes were characterized by a significant burden difference between healthy controls and ExAC.

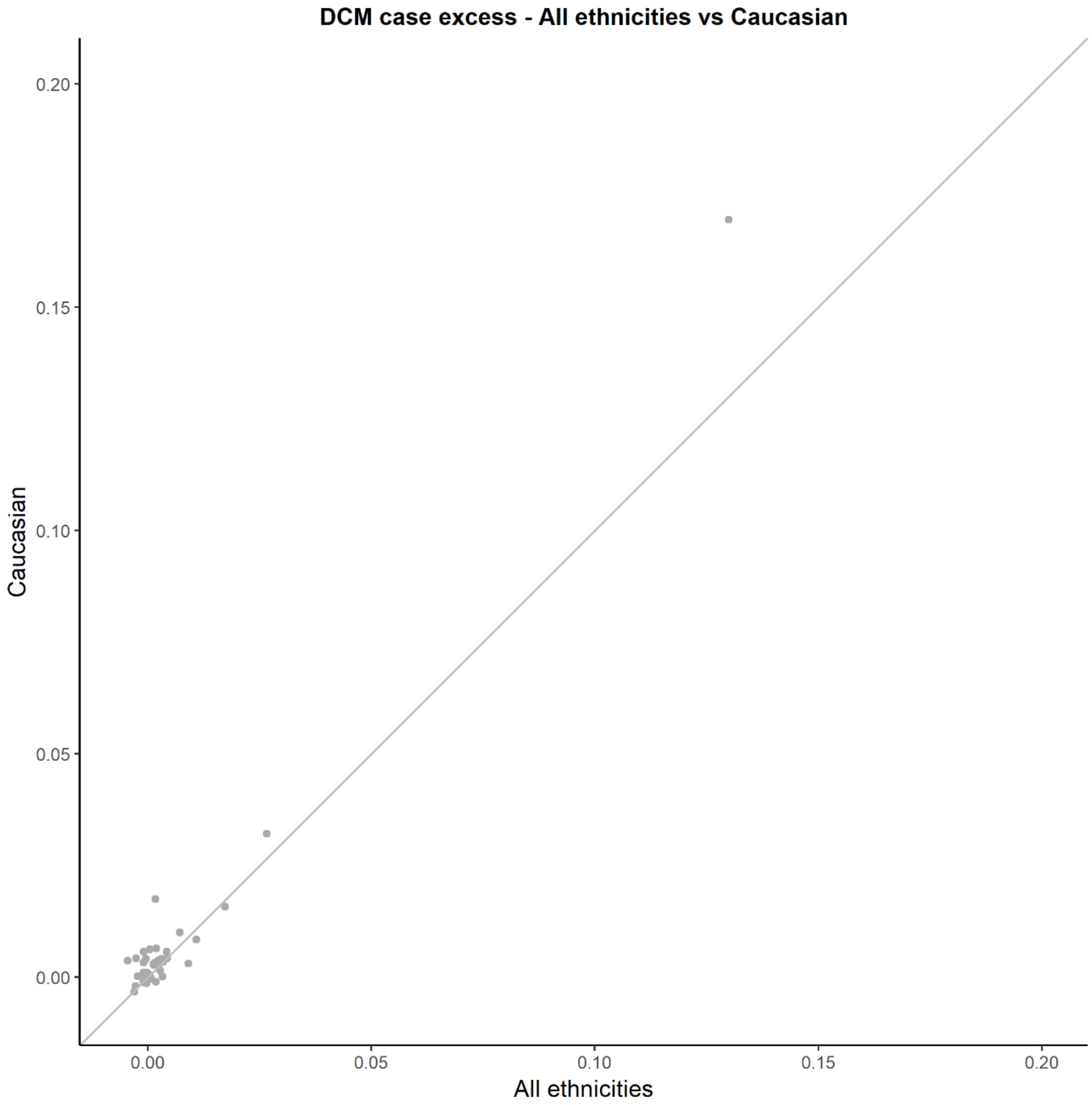

Supplemental Figure 6 — Comparison of burden testing results (excess frequency in DCM vs ExAC) obtained including samples of any ethnic group (all DCM patients vs the whole ExAC dataset) compared to results obtained on Caucasian cases and controls (self-reported Caucasian DCM patients vs Non-Finnish Europeans individuals in ExAC). The Pearson correlation coefficient between the two variables was 0.975 and no additional significant associations were detected when restricting the analysis to samples of Caucasian/Non-Finnish Europeans descent.

SUPPLEMENTAL REFERENCES

1. Maceira AM, Prasad SK, Khan M, Pennell DJ. Normalized left ventricular systolic and diastolic function by steady state free precession cardiovascular magnetic resonance. *J Cardiovasc Magn Reson*. 2006;8:417–426.

2. Henry WL, Gardin JM, Ware JH. Echocardiographic measurements in normal subjects from infancy to old age. *Circulation*. 1980;62:1054–1061.

3. bcl2fastq Conversion Software [Internet]. [cited 2018 Feb 27];Available from: https://support.illumina.com/sequencing/sequencing_software/bcl2fastq-conversion-software.html

4. Babraham Bioinformatics - FastQC A Quality Control tool for High Throughput Sequence Data [Internet]. [cited 2015 Dec 4];Available from: http://www.bioinformatics.babraham.ac.uk/projects/fastqc/

5. Schmieder R, Edwards R. Quality control and preprocessing of metagenomic datasets. *Bioinformatics*. 2011;27:863–864.

6. Li H, Durbin R. Fast and accurate short read alignment with Burrows-Wheeler transform. *Bioinformatics*. 2009;25:1754–1760.

7. Picard Tools - By Broad Institute [Internet]. [cited 2015 Dec 4];Available from: http://broadinstitute.github.io/picard/

8. McKenna A, Hanna M, Banks E, Sivachenko A, Cibulskis K, Kernytsky A, Garimella K, Altshuler D, Gabriel S, Daly M, DePristo MA. The Genome Analysis Toolkit: A MapReduce framework for analyzing next-generation DNA sequencing data. *Genome Res*. 2010;20:1297–1303.

9. Guo MH, Plummer L, Chan Y-M, Hirschhorn JN, Lippincott MF. Burden Testing of Rare Variants Identified through Exome Sequencing via Publicly Available Control Data. *Am J Hum Genet*. 2018;103:522–534.

10. Champely S. pwr: Basic Functions for Power Analysis. R package version 1.2-2. [Internet]. 2018;Available from: https://CRAN.R-project.org/package=pwr

11. Ihaka R, Gentleman R. R: A Language for Data Analysis and Graphics. *Journal of Computational and Graphical Statistics*. 1996;5:299–314.

12. Hildebrandt M, Bender R, Gehrmann U, Blettner M. Calculating confidence intervals for impact numbers. *BMC Med Res Methodol*. 2006;6:32.

13. Walsh R, Thomson KL, Ware JS, Funke BH, Woodley J, McGuire KJ, Mazzarotto F, Blair E, Seller A, Taylor JC, Minikel EV, Exome Aggregation Consortium null, MacArthur DG, Farrall M, Cook SA, Watkins H. Reassessment of Mendelian gene pathogenicity using 7,855 cardiomyopathy cases and 60,706 reference samples. *Genet Med*. 2017;19:192–203.

14. Pugh TJ, Kelly MA, Gowrisankar S, Hynes E, Seidman MA, Baxter SM, Bowser M, Harrison B, Aaron D, Mahanta LM, Lakdawala NK, McDermott G, White ET, Rehm HL, Lebo M, Funke BH. The landscape of genetic variation in dilated cardiomyopathy as surveyed by clinical DNA sequencing. *Genet Med*. 2014;16:601–608.
